# Effect of a short-term dietary supplementation with phytosterols, red yeast rice or both on lipid pattern in moderately hypercholesterolemic subjects: a three-arm, double-blind, randomized clinical trial

**DOI:** 10.1186/s12986-017-0214-2

**Published:** 2017-09-25

**Authors:** Arrigo F.G. Cicero, Federica Fogacci, Martina Rosticci, Angelo Parini, Marina Giovannini, Maddalena Veronesi, Sergio D’Addato, Claudio Borghi

**Affiliations:** Medical and Surgical Sciences Department, University of Bologna, Via Albertoni 15, 40138 Bologna, Italy

**Keywords:** Monacolins, LDL-cholesterol, Phytosterols, Red yeast Rice, Nutraceuticals, Clinical trial

## Abstract

**Background:**

Phytosterols and red yeast rice are largely studied cholesterol-lowering nutraceuticals, respectively inhibiting the bowel absorption and liver synthesis of cholesterol. Our aim was to test the effect on lipid profile of phytosterols, red yeast rice and their association.

**Methods:**

We performed a three parallel arms, double blind, clinical trial randomizing 90 moderately hypercholesterolemic subjects to treatment with phytosterols 800 mg (group 1), red yeast rice standardized to contain 5 mg monacolins from *Monascus purpureus* (group 2), or both combined nutraceuticals (group 3).

**Results:**

After 8 weeks of treatment, in group 1 no significant variation of lipid parameters has been detected. In group 2 a significant reduction (*p* < 0.001) of LDL-Cholesterol (−20.5% vs. baseline) and Apolipoprotein B (−14.4% vs. baseline) as it occurred in group 3 (LDL-Cholesterol vs. baseline: −27.0%, Apolipoprotein B vs. baseline: -19.0%) (*P* < 0.001). LDL-Cholesterol and Apolipoprotein B changes were significantly different comparing group 2 with group 1 (*P* < 0.05), and group 3 with group 1 (*P* < 0.05). LDL-Cholesterol change was also significantly higher in group 3 than in group 2 (*P* < 0.05).

**Conclusion:**

The association of phytosterol and red yeast rice seems to have additive cholesterol lowering effect, reaching a clinically significant LDL-Cholesterol reduction in mildly hypercholesterolemic patients.

**Trial registration:**

ClinicalTrial.gov ID: NCT02603276, Registered 27/08/2015.

## Background

A relatively large number of dietary supplements and nutraceuticals have been studied for their supposed or demonstrated ability to reduce cholesterolemia in humans [1]. Nutraceuticals are found in a mosaic of products emerging from the food industry, the herbal and dietary supplement market and pharmaceutical industry. They may range from isolated nutrients, herbal products, dietary supplements that mainly includes soluble fibers, phytosterols, soy proteins, omega 3 polyunsaturated fatty acids, monacolins, policosanols, berberine and garlic extracts [2].

Phytosterols (plant sterols and stanols) are natural constituents of cell membrane of plants. Their role in plants is similar to that of cholesterol in humans [3]. Structurally, they are also similar to cholesterol, with only minor differences in relative position of ethyl and methyl groups. This similarity may suggest their capacity on cholesterol lowering [4]. Moreover, several clinical trials have consistently shown that intake of 2–3 g/day of plant sterols is associated with significant lowering (between 4 and 15%) of LDL-cholesterol [2, 5–7]. Total cholesterol is also reduced to similar extent, while it is uncertain whether the use of phytosterols has any beneficial effect on triglyceride levels [8, 9]. Based on the available data, the European Food Safety Agency (EFSA) accepted a health claim for the phytosterols LDL-cholesterol lowering effect [10].

The red yeast rice is a dietary supplement made by fermenting the yeast, *Monascus purpureus*, over rice. Monascus yeast produces a family of substances called monacolins, including monacolin K. Monacolins act as reversible inhibitors of the 3-hydroxy-3-methyl-glutaryl-coenzyme A (HMG-CoA) reductase, the key enzyme in the cholesterol biosynthesis. In addition to the inhibitors of HMG-CoA reductase, red yeast rice has been found to contain sterols (β-sitosterol, campesterol, stigmasterol, and sapogenin), isoflavones and isoflavone glycosides, and monounsaturated fatty acids [11], all capable of lowering LDL-Cholesterol [12]. Recently, red yeast rice has been used to lower LDL cholesterol levels in patients who have had to discontinue the use of statin medication due to muscle pains, confirming its good tolerability, both in term of changes in biochemical parameters and muscle pain severity [13]. Red yeast rice is indeed similar to pravastatin in term of LDL-C reduction (27% vs. 30%, *p* > 0.05), but is associated with a lower rate of withdrawn from the treatment [14]. Importantly, based on the available data, EFSA accepted a health claim on the LDL-C red yeast rice reducing effect [15].

In this context, the aim of this three-arm, double-blind, randomized clinical trial was to comparatively test the effect on lipid pattern of 8 weeks treatment with phytosterols, red yeast rice or both nutraceuticals in moderately hypercholesterolemic subjects, as well as their tolerability.

## Methods

### Study design

This three parallel arms, double blind, randomized clinical trial was carried out in 90 moderately hypercholesterolemic subjects, non-smokers, pharmacologically untreated, in primary prevention for cardiovascular diseases, consecutively enrolled in the ambulatory service of cardiovascular disease prevention in the Medical and Surgical Sciences Department of the University of Bologna.

Inclusion criteria were age between 18 and 70, and LDL-Cholesterol level between 130 and 190 mg/dL, confirmed in at least two sequential checks before to sign the consent form.

Exclusion criteria were:

- Personal history of cardiovascular disease nor risk equivalents
- TG > 400 mg/dL and/or HDL-C < 35 mg/dL
- Obesity (BMI > 30 kg/m2)
- Assumption of lipid-lowering drugs or drugs affecting lipid metabolism
- Known thyroid, liver, renal or muscle diseases

The study was fully conducted in accordance with the Declaration of Helsinki, its protocol was approved by the Ethical Committee of the University of Bologna, and informed consent was obtained from all patients before the inclusion in the study (Clinicaltrial.gov ID NCT02603276).

At enrollment visit (T-1), patients were given standard behavioral and qualitative (not quantitative) dietary suggestions to correct unhealthy habits. Standard diet advice was given by a dietitian and/or specialist doctor. Dietitian and/or specialist doctor periodically provided instruction on dietary intake recording procedures as part of a behavior modification program and then later used the subject’s food diaries for counseling. In particular, subjects were instructed to follow general indication of a Mediterranean diet, avoiding excessive intake of dairy products and red meat derived products during the study, maintaining overall constant dietary habits. Individuals were also generically encouraged to increase their physical activity by walking briskly for 20 to 30 min, 3 to 5 times per week, or by cycling.

### Treatments

After 2 weeks of diet and physical activity (T0), patients were allocated to treatment with an indistinguishable liquid sticks containing three different nutraceuticals: 1) phytosterols 800 mg, 2) red yeast rice standardized to contain 5 mg monacolins from *Monascus purpureus* (Dif1stat®) [16, 17]*,* 3) both combined nutraceuticals (kindly offered by Difass International S.r.l., Prato, Italy). The red yeast rice extract used was certified to be highly purified in monacolins, without chromatographically detectable levels of dehydromonacolins, decalin derivatives, and contaminants. The phytosterol dose was chosen on the basis of the minimal efficacious dose identified by the meta-analysis of randomized clinical trial performed by Demonty et al. [18].

The treatment has then continued for 8 weeks. Clinical and laboratory data have been obtained at the baseline (T0) and at the end of the trial (T1). Randomization was done using a drawing of envelopes containing randomization codes prepared by an independent statistician and specific software. The envelopes were then further mixed and distributed to the investigators who assigned the randomization code in a progressive way to the enrolled subjects. A copy of the code was provided only to the person responsible of performing the statistical analysis.

Throughout the study, we instructed patients to take the new product first dose on the day after they were given the study product in a blinded box. At the same time, all unused products were retrieved for inventory. Product compliance was assessed by counting the number of product doses returned at the time of specified clinic visits.

### Assessments

All plasma parameters were obtained after a 12-h overnight fast. Venous blood samples were drawn by a nurse in all patients between 8:00 a.m. and 9:00 a.m. Serum used was centrifuged at 3000 g for 15 min at ambient temperature. Immediately after centrifugation, the samples were frozen and stored at −80 °C for no more than 3 months. The following parameters were evaluated via standardized methods [19, 20]: total cholesterol (TC), high-density lipoprotein-cholesterol (HDL-C), triglycerides (TG), LDL-Cholesterol (LDL-C), apolipoprotein AI (apoAI), apolipoprotein B100 (apoB), glucose, creatinine, serum uric acid, liver transaminases, gamma-Glutamyl Transferase, and Creatinin-Phosfo-Kinase (CPK). All measurements were performed by trained personnel in the Lipid Clinic laboratory of the Medicine and Surgery Sciences Department, by the S.Orsola-Malpighi University Hospital.

### Statistical analysis

Data have been analyzed using intention to treat by mean of the Statistical Package for Social Science (SPSS) 19.0, version for Windows. The sample size suggested to detect a mean difference of 5% between treatments in term of LDL-reduction, with a power of 0.90 and an alpha error of 0.05, was of at least 20 subjects per group. As per protocol, we decided a priori to check the efficacy of treatments in subjects assuming at least the 90% of the tested products doses foreseen by the trial design. Normally distributed baseline characteristics of the population have been compared using Student’s *t* test and χ^2^ test followed by Fisher’s exact test for categorical variables. Between group difference was assessed by the ANOVA followed by the Tukey’s post-hoc test. A linear regression analysis was carried out to detected an eventual correlation between basal LDL-C level and percentage LDL change. All data are expressed as means and SD. A *p* level of <0.05 was considered significant for all tests.

## Results

Ten patients in the phytosterol group, 8 in the red yeast rice one, and 7 in the red yeast rice plus phytosterols assumed less than the 90% of doses foreseen by the protocol, so that they were excluded from the analysis. No patient dropped out from the study because of adverse events.

Enrolled patients were age- and sex- matched. The baseline characteristics of patients assigned to the three different treatments (phytosterols, red yeast rice or combined) were similar and no significant differences were observed regarding the studied parameters (Table 1).

**Table 1:** Characteristics of the patients enrolled after the stabilization diet period by treatment group

	Phytosterols (N. 20; M: 8. F: 12)		Red Yeast Rice (N. 22; M: 11. F: 11)		Phytosterols + Red Yeast Rice (N. 23; M: 12. F: 11)	
	Mean	SD	Mean	SD	Mean	SD
Age (years)	53.6	12.3	49.9	14.5	53.0	11.2
Total Energy Intake (kcal)	2284	139	2310	137	2271	148
Fat dietary intake (% on total energy)	26.9	2.8	27.2	2.9	27.5	3.0
Saturated fat intake (Fat dietary intake (% on total energy)	11.1	1.4	10.2	1.3	10.9	1.3
Protein intake (% on total energy)	18.1	2.1	17.9	2.2	17.2	2.3
Carbohydrate intake (% on total energy)	51.4	3.2	50.6	3.1	51.2	4.0
Cholesterol intake (mg)	196.4	9.3	190.9	9.1	198.9	10.1
Waist circumference (cm)	88.9	10.4	85.1	8.9	90.2	12.8
Body Mass Index (Kg/m2)	25.0	2.6	25.2	2.9	25.6	3.4
Systolic blood pressure (mmHg)	130.7	17.2	122.5	15.8	124.4	17.1
Diastolic blood pressure (mmHg)	79.4	12.3	76.7	14.0	80.0	10.6
Pulse pressure (mmHg)	51.3	10.9	45.7	9.8	44.4	14.3
Total Cholesterol (mg/dl)	220.9	26.7	235.8	23.0	237.9	27.7
Triglycerides (mg/dl)	136.4	75.7	121.8	53.5	109.4	44.3
HDL-Cholesterol (mg/dl)	46.7	13.1	53.1	11.4	48.9	12.8
LDL-Cholesterol (mg/dl)	146.8	27.3	158.3	21.1	167.1	25.1
ApolipoproteinA1 (mg/dl)	140.9	20.6	158.1	22.4	148.6	29.4
ApolipoproteinB100 (mg/dl)	92.4	17.9	98.4	15.9	101.4	19.1
Glucose (mg/dl)	91.6	8.6	88.6	10.9	90.3	13.2
Uricemia (mg/dl)	5.4	1.1	4.8	1.3	5.1	1.14
Creatinine (mg/dl)	0.98	0.14	0.92	0.13	0.98	0.16
eGFR (CKD-EPI equation) (ml/min/1.73 m2)	77.9	15.0	81.9	16.6	79.1	11.9
AST (U/l)	21.3	4.6	20.0	5.4	19.5	4.9
ALT (U/l)	25.2	9.7	21.6	8.6	22.0	9.6
Gamma-Glutamyl Transferase (U/l)	29.2	27.7	22.8	16.9	18.3	7.8
Creatine Posphokinase (U/l)	125.1	86.8	136.4	66.8	118.5	73.8

From the randomization visit (T0) to the end of the study (T1), the enrolled subjects maintained overall a similar dietary pattern, without significant change in total energy, total cholesterol and total saturated fatty acid intake.

**Fig. 1:**
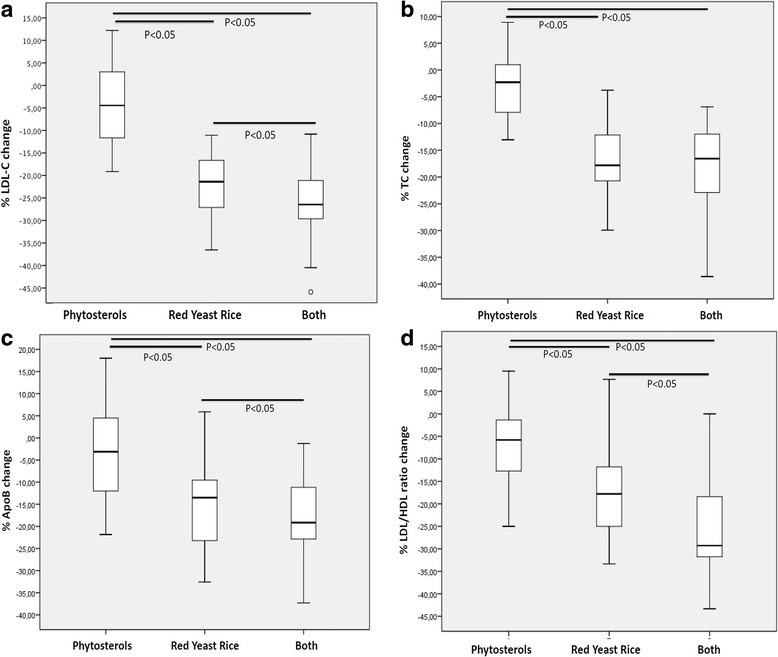
Percentage change of LDL-cholesterolemia (**a**), Total Cholesterol (**b**), Apolipoprotein B (**c**) and LDL to HDL ration (**d**) in the three treatment groups versus baseline

After 8 weeks of treatment, we noted in group 1 (treated with phytosterols 800 mg only) a slight but not significant improvement vs baseline levels of the lipid parameters measured; in group 2 (treated with red yeast rice standardized to contain 5 mg monacolins) we found a significant reduction (*p* < 0.001) of TC levels (TC vs. baseline: −16.1 ± 2.3%), of LDL-C levels (LDL-C vs. baseline: −20.5 ± 3.4%) and of Apo B 100 levels (ApoB vs. baseline: −14.4 ± 2.1%); in group 3 (treated with both combined nutraceuticals) we also observed a significant reduction (*p* < 0.001) of TC levels (TC vs. baseline: −18.5 ± 3.1%), of LDL-C levels (LDL-C vs. baseline: −23.0 ± 3.5%) and of Apo B levels (ApoB vs. baseline: −19.0 ± 3.3%). There was a significant change (*P* < 0.05) in TC, LDL-C and ApoB levels after the treatment between group 2 and group 1, and a significant change (*P* < 0.05) in TC, LDL-C and ApoB levels after the treatment between group 3 and group 1 (Table 2). Moreover, we detected a significant reduction of LDL-C levels (*p* < 0.05) in group 3 than in group 2 (LDL-C group 3 vs LDL-C group 2: −6.5 ± 1.2%) (Fig. 1). A proportional effect has been also observed as it regards apoB level and LDL to HDL ratio (Fig. 1).

**Table 2:** Changes in lipids, lipoproteins and their ratios from the end of the stabilization diet (T0) to the end of the experimental treatment period (T1)

	Phytosterols (Group 1; M: 8, F: 12)		P vs. baseline	Red Yeast Rice (Group 2; M: 11, F: 11)		P vs. baseline	Phytosterols + Red Yeast Rice (Group 3; M: 12, F: 11)		P vs. baseline
	Mean	SD		Mean	SD		Mean	SD	
Δ TC (mg/dl)	−5.4	15.2	0.127	−38.1*	16.7	<0.001	−44.2*	25.8	<0.001
Δ TG (mg/dl)	−0.8	59.0	0.938	−16.6	54.2	0.164	+4.8	34.3	0.536
Δ HDL-C (mg/dl)	+1.0	5.5	0.430	+2.2	5.7	0.076	+0.1	7.1	0.951
Δ LDL-C (mg/dl)	−6.6	15.7	0.075	−32.5*	12.5	<0.001	−45.2*°	20.2	<0.001
Δ TC/HDL-C	−0.2	0.5	0.109	−0.6*	−0.3	<0.001	−0.8*	0.6	<0.001
Δ LDL-C/HDL-C	−0.2	0.4	0.050	−0.5*	−0.3	<0.001	−0.9*°	0.5	<0.001
Δ Apo B100 (mg/dl)	−3.0	11.8	0.271	−14.2*	11.0	<0.001	−19.3*°	13.1	<0.001
Δ ApoA1 (mg/dl)	+2.6	17.7	0.513	+6.7	19.5	0.122	+1.7	20.7	0.718
Δ Apo B100/ApoAI	−0.03	0.07	0.095	−0.1*	0.07	<0.001	−0.1*	0.08	<0.001

*TC* Total Cholesterol, *TG* Triglycerides, *Apo* Apolipoprotein

*p < 0.05 Vs. Phytosterols (Group 1); ° *p* < 0.05 Vs. Red Yeast Rice (Group 2)

The observed percentage LDL change was proportional to the baseline LDL-level (B = −0.149, 95% CI -0.283 - -0.016, *p* = 0.029). A similar trend has been observed also dividing the patient sample by treatment.

## Discussion

Several studies have shown the lowering effect on lipid profile of nutraceuticals and functional foods, as reducing TC, TG, and LDL-C, while elevating HDL-C [1, 2, 21]. We performed a three parallel arms, double blind, placebo-controlled, randomized clinical trial to study the specific effect of phytosterols, red yeast rice and both combined nutraceuticals on lipid parameters in moderately hypercholesterolemic subjects, non-smokers, pharmacologically untreated, in primary prevention for cardiovascular diseases. We evaluated the tolerability and effectiveness of three different treatments, monitoring clinical and laboratory data at baseline and after 8 weeks at the end of the trial. It is well known that the role of phytosterols in plants is similar to that of cholesterol in humans [3]. This similarity may suggest their capacity of cholesterol absorption inhibition and lower LDL-C levels [2, 5]. Meta-analyses have shown that consuming approximately 2.5 g of phytosterols per day lowers serum LDL-C levels up to 10%, with little additional benefit achieved at higher intakes [22]. Epidemiological studies in the UK, Sweden and China observed that naturally occurring dietary plant sterol intake is inversely related to plasma TC and LDL-C levels [23–25]. In our study, we noted a slight reduction of TC and LDL-C levels subjects treated with phytosterols 800 mg vs baseline levels, but this result was not statistically significant. This was probably related to the use of a minimal effective dose [19] in a relatively small patient sample, already under correct dietary habits. In particular, the small sample could have reduced the possibility to observe a significant effect, since the phytosterol effect is strongly affected by individual genetic characteristics [26]. Instead, subjects treated with red yeast rice experienced an important reduction of TC, LDL-C and ApoB 100 levels vs baseline levels. On the other hand, the effectiveness of red yeast rice in lowering LDL cholesterol is well known [12–14, 27], being its mechanism of action the inhibition of the HMG-CoA reductase enzyme in the liver. In fact, Monacolin K, the most concentrated monacolin in red yeast rice, is lovastatin [11], a commonly prescribed as lipid-lowering drug. Finally, the association of phytosterols to red yeast rice magnified the results obtained with red yeast rice alone, reaching a further significant reduction of the LDL-C levels, thus showing that the tested phytosterol dose could improve the already significant cholesterol-lowering effect of red yeast rice per se. This is partly in contrast with a previous trial carried out by Becker et al., where phytosterol supplementation did not improve the LDL-C lowering effect of red yeast rice. However in this study there was a high drop-out and non-compliance rate, maybe related to the need to assume 10 pills per day during the trial [28].

In our trial, the LDL-C reduction achieved is near to that 40 mg/dL reduction in LDL-C estimated to be associated with a corresponding 22% reduction in cardiovascular mortality and morbidity [29]. These results were partly expected, because the mechanism of action of red yeast rice and phytosterols should be additive or synergistic, [30] representing the natural alternative to the synergistic association of statins with ezetimibe [31].

Our study has some relevant limitations. The first one is the relatively low number of subjects investigated per treatment group, related to the exclusion of those with an unexpected low compliance to the treatment. However, the study was sufficiently powered even with the sample including the compliant patients only, and the low compliance was not related to adverse events. The second one is the lack of a pure placebo group: in effect, we compared consolidated treatments and their association, where phytosterols and red yeast rice efficacy had been already largely evaluated in numerous double-blind placebo-controlled randomized clinical trials (EFSA Panel on Dietetic Products, Nutrition and Allergies [15]; EFSA Panel on Dietetic Products, Nutrition and Allergies [10]). Anyway, the absence of a placebo group makes the conclusions of this study preliminary and with the need to be further confirmed before reaching an adequate clinical applicability. The third one is the lack of measurement of markers of cholesterol absorption and synthesis. Finally, the study was relatively short, so that we do not know if the observed effect could be confirmed in the long-term. However, being the mechanism of action of monacolins and phytosterols the ones of drugs already proven to maintain their efficacy over years, we have no reason to doubt that this evidence could be translated to the tested nutraceuticals.

However, the dietary supplementation of red yeast rice and/or phytosterols are suggested by international guidelines and statements of some scientific societies [32, 33] and our data further support these suggestions.

## Conclusions

In conclusion, on the basis of our trial it seems that the additive lipid-lowering effect of phytosterols and red yeast rice can improve lipid parameters with a good short-term tolerability, and could represent a therapeutic alternative in patients in primary prevention with moderate hypercholesterolemia.

## Abbreviation

ApoAI: Apolipoprotein AI
ApoB: Apolipoprotein B100
CPK: Creatinin-Phosfo-Kinase
HDL-C: High-density lipoprotein-cholesterol
LDL-C: LDL-Cholesterol
TC: Total cholesterol
TG: Triglycerides
