# Correction to: Effect of a short-term dietary supplementation with phytosterols, red yeast rice or both on lipid pattern in moderately hypercholesterolemic subjects: a three-arm, double-blind, randomized clinical trial

**DOI:** 10.1186/s12986-018-0280-0

**Published:** 2018-06-20

**Authors:** Arrigo F. G. Cicero, Federica Fogacci, Martina Rosticci, Angelo Parini, Marina Giovannini, Maddalena Veronesi, Sergio D’Addato, Claudio Borghi

**Affiliations:** Medical and Surgical Sciences Department, University of Bologna, Via Albertoni 15, 40138 Bologna, Italy

## Correction

Following publication of the original article [1], the authors reported a typo in the article category.

The misspelled article category in the original article is:

- REAEARCH

The correct article category is spelled as:

- RESEARCH

An error was introduced during the production process; the article category should be RESEARCH and not REAEARCH.
